# A high quality Aotearoa New Zealand dietary pattern adapting a Mediterranean diet for metabolic health: a feasibility study

**DOI:** 10.1186/s40795-023-00805-x

**Published:** 2023-12-08

**Authors:** Amber Parry-Strong, Richard Gearry, Troy L. Merry, Mark Weatherall, Cheryl Davies, Anna Worthington, Rhiane Bishop, Summer Rangimaarie Wright, Fiona E. Lithander, Meika Foster, Jeremy Krebs

**Affiliations:** 1 Centre for Endocrine, Diabetes and Obesity Research, Te Whatu Ora New Zealand Capital, Coast and Hutt Valley, PO Box 7902, Wellington, New Zealand; 2 The Liggins Institute, University of Auckland, Auckland, New Zealand; 3 Department of Medicine, University of Otago, Christchurch, New Zealand; 4 Department of Nutrition and Dietetics, School of Medical Sciences, University of Auckland, Auckland, New Zealand; 5 Department of Medicine, University of Otago, Wellington, PO Box 7343, Wellington South, 6242 New Zealand; 6 Tū Kotahi Māori Asthma and Research Trust, Kōkiri Marae, Lower Hutt, New Zealand; 7 Edible Research Ltd, RD2 Kaiapoi, Ohoka Christchurch 7692 New Zealand; 8 New Zealand National Science Challenge High Value Nutrition, Liggins Institute, University of Auckland, Building 505, 85 Park Road, Auckland, 1023 New Zealand

**Keywords:** Metabolic syndrome severity score, Dietary pattern, Cardio-metabolic risk, Food delivery, Family intervention

## Abstract

**Aim:**

To assess the feasibility of a family-based dietary intervention study using a meal kit home delivery service, in people at risk of cardio-metabolic disease.

**Methods:**

A 12-week dietary intervention feasibility study of adults (termed the index participants) at increased risk of metabolic and cardiovascular disease, enriched for Māori who are indigenous New Zealanders. The study sample also included the household/whānau members living with the index participant. All participants received a 12 week intervention using weekly home delivery of meal kits and groceries consistent with a Mediterranean dietary pattern. Outcomes were the metabolic syndrome severity score (MetSSS); feasibility and acceptability of the intervention; dietary intake; and other clinical and anthropometric measures.

**Results:**

There were 29 index participants recruited and in addition, 50 household/whānau members took part in the feasibility study. The mean (SD) household/whānau size was 3.45 (1.4) people, and the mean (SD) number of people in each household/whānau who participated in the study was 2.84 (1.2). The feasibility of intervention to households/whānau was proven in this context. The mean (SD) change in MetSSS was 0.03 (0.33), *N* = 27, *P* = 0.69 and there was a statistically significant decrease in body weight of 1.37 kg (95% CI 0.11 to 2.62), *p* = 0.034. The food deliveries were well received, the dinner kits more so than the grocery items.

**Conclusion:**

It is feasible to recruit individuals and households/whānau to a family-based dietary intervention. Use of a meal kit home delivery service to provide food which is consistent with the intervention dietary pattern was well received. This feasibility study identified improvements to be made such as nutrition behaviour change support, more variety in food provided, more recipes, and better matching of food quantity to family size.

**Trial registration:**

ANZCTR—ACTRN12621000856819p registered 2.JUN.2021 https://www.anzctr.org.au/Trial/Registration/TrialReview.aspx?id=382021&isReview=true

**Supplementary Information:**

The online version contains supplementary material available at 10.1186/s40795-023-00805-x.

## Background

Diet is one of the most important modifiable risk factors for cardio-metabolic disease [1]. Epidemiological studies have shown that a diet of whole foods rich in mono and poly unsaturated fats, with minimal saturated fat, such as the Mediterranean diet (MD), is associated with lower rates of cardiovascular disease (CVD) and diabetes [2]. The MD is characterised by a high vegetable consumption with regular consumption of nuts, seeds, fruit, fish, legumes, dairy products and wholegrain cereals [3]. Intervention studies to promote a Mediterranean dietary pattern in non-Mediterranean multi-ethnic populations are acceptable [4] and have shown improvements in CVD risk factors and metabolic health [5, 6]. The PREDIMED study in Spain compared 3 arms – an MD with olive oil, and MD with nuts and a control diet. Those randomised to the MD with nuts demonstrated a diabetes incidence of 11.0% (5.9–16.1) compared with 17.9% (11.4–24.4) in the control group, and the two diet arms pooled showed a 52% reduction in progression to diabetes compared with control, in participants at increased risk of CVD after 4-years of follow up [7]. As a wide range of foods align with the key nutritional parameters of a Mediterranean dietary pattern, the MD can be tailored and applied in different cultural settings and seasons. Adherence to a Mediterranean dietary pattern in New Zealand (NZ) is currently low [8].

Aotearoa/New Zealand continues to have a high prevalence of obesity, CVD and type 2 diabetes mellitus (T2DM) with an estimated rate of T2DM in 2021 of 41.5 (95% CI: 41.4, 41.7) per 1000 population [9, 10]. Māori (Indigenous New Zealanders) and Pacific peoples carry a greater burden of these conditions than other ethnicities with rates of T2DM at 70.1 and 118.8 per 1000 respectively [11]. The metabolic syndrome (MetS) refers to the clustering of several risk factors, including hypertension, central adiposity, dyslipidemia and insulin resistance, which identifies individuals at greater risk of CVD and T2DM [12]. MetS is commonly classified in a dichotomous fashion, as having or not having MetS. An alternative approach, which gives a continuous and more nuanced description, is the Metabolic Syndrome Severity Score (MetSSS) which quantifies the value of MetS latent factors for an individual, and the resulting score behaves like a Z-score in that it is normally distributed in a population. The MetSSS has been shown to predict future cardio-metabolic disease and can be modified by lifestyle including diet and exercise, and pharmacological interventions to corresponding change in cardio-metabolic disease risk. It is therefore a useful tool in assessing the impact of a dietary intervention on metabolic health [13]. It has been validated in a New Zealand population and has been shown to be responsive to diet and lifestyle interventions in people with pre-diabetes [13–15].

Cardio-metabolic risk is often congruous among household/whānau members as dietary patterns tend to be similar throughout the household [16, 17]. Children can be susceptible to poor dietary habits as they have less control over what they consume, which can lead to an increased risk of metabolic abnormalities in later life [18]. Dietary restriction is not ideal for children as it can lead to negative relationships with food [17], so creating a positive environment around dietary patterns for the whole whānau should be emphasised. Interventions targeting healthy behaviour in parents have shown improvements in their children’s BMI z-score and dietary behaviour patterns [19]. A large cohort study found that children who adhered to the MD at 4 years old experienced lower rates of obesity and abdominal adiposity at age 8 years [20].

This is a feasibility study for a future dietary intervention trial. It is recommended that healthcare interventions should undergo feasibility testing prior to evaluation in a full-scale trial [21]. This study aimed firstly to test the feasibility and acceptability of providing a dietary pattern consisting of food products based on the MD for individuals and their whānau/household. Secondly, it sought to test the feasibility of recruiting and retaining Māori participants, through an Indigenous Māori Health Centre. The third aim was to collect MetSSS data, and in particular an estimate of standard deviation, to enable a realistic sample size for the future trial. Further, this study seeks to determine the recruitment rates for individuals to participate and the acceptability and uptake of family members to be included in the study.

## Methods

This study was a 12-week non-randomised intervention of adults at increased risk of metabolic and cardiovascular disease (described as “index individuals”), enriched for Māori participants. The study population also included the household/whānau members living with the index individual. It was conducted at the Centre for Endocrine, Diabetes and Obesity Research (CEDOR) in Wellington, and at Kōkiri Marae in Lower Hutt, New Zealand. As part of supporting Māori research capability building, Tū Kotahi Māori Asthma and Research Trust was supported to lead the delivery of the intervention through Kōkiri Marae Health and Social Services. The trial was registered with the Australian New Zealand Clinical Trials Registry: ACTRN12621000856819p. The study was approved by the New Zealand Health and Disability Ethics Committee: 21/NTA/113.

### Participants

The study aimed to recruit 30 index individuals and their household/whānau. Index participants were recruited if they met the following inclusion criteria: Adults aged 18–70 years, metabolic syndrome (defined as MetSSS > 3.5), and at least one (and up to eight) household/whānau members agreeing to participate. A cut-off MetSSS of 3.5 was chosen as it reflected a point above which cardio-metabolic disease was more likely in the New Zealand population [14]. Individuals were not eligible to participate if they met any of the exclusion criteria: previous bariatric surgery; pre-existing Type 1 or Type 2 diabetes (two HbA1c results ≥ 50 mmol/mol minimum three months apart [22]); stage 4/5 renal disease; severe food allergy; current pregnancy or breastfeeding, or planning to conceive during the study; unstable body weight (active weight loss/gain > 5 kg in previous three months); gastrointestinal disorder that alters the digestion and absorption of nutrients (e.g. coeliac disease, ulcerative colitis, Crohn’s disease, an ileostomy or colostomy);; medications that modify blood sugar levels.

### Recruitment

Participants were recruited at two sites. Tū Kotahi, which delivers health and social services through Kōkiri Marae, aimed to recruit 10 Māori participants and their whānau through their existing networks. The other 20 participants were to be recruited through CEDOR by advertising to the general public through relevant email lists. All those who expressed interest in the study were assessed for eligibility using a two-step process. First, a telephone screening call including the calculation of the Australian Type 2 Diabetes Risk Assessment Tool (AUSDRISK) score, a screening tool that identifies people at risk of type 2 diabetes [23]. This was used to maximise the likelihood that those invited to the second part of screening would meet the study inclusion criteria. Individuals who had an AUSDRISK score ≥ 12 were then invited to attend the research centre in person for questionnaires and measurement of variables to calculate their MetSSS. Those who met all of the inclusion were enrolled in the study. The household/whānau members were then also invited to attend at visit 2. Informed consent was obtained from all the participants and their legal guardians, and assent from children under the age of 16 years, prior to any data collection.

### Study visits

**Fig. 1:**
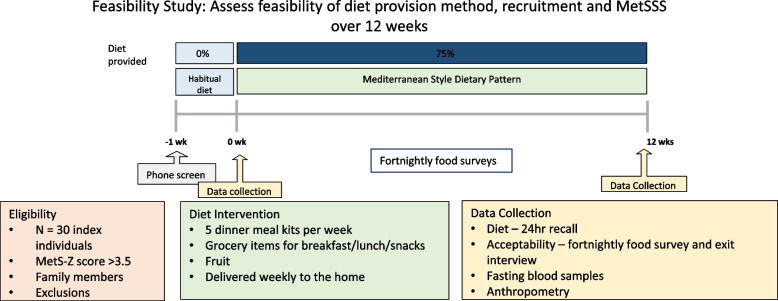
Flow chart of study

Participants attended study visits at baseline and 12 weeks (Fig. 1). Both visits comprised anthropometric measurements, fasting venous blood tests and a dietary intake questionnaire (Table 1). Medical conditions and history were recorded at baseline and current medications were recorded at baseline and 12 weeks.

**Table 1:** Visit measures and questionnaires

Measurement	Measurement tool / method used
Height	Fixed stadiometer (cm) without shoes
Weight	Bioimpedance scales measured with no shoes and minimal clothing on a hard surface
Waist circumference	Measured at the level of the iliac crest
Blood Pressure	Mindray VS900 automatic BP machine (mmHg)
HbA1c	HPLC—(D-100; Biorad, CA, USA)
Lipid profile	Roche Cobas Enzymatic Colorimetric (mmol/L)
Fasting glucose	Roche Cobas, Hexokinase (mmol/L)
Dietary intake	24 h dietary recall – Intake24 online (https://intake24.co.uk/)
Acceptability	Fortnightly Survey, Exit Interview

### Dietary intervention

Participants were provided with a range of foods that are consistent with an adapted version of the Mediterranean dietary pattern and nuts were provided instead of olive oil [4]. Foods provided were assessed for their nutrient content and provided at a level that would meet the threshold for 75% of the nutrient reference values required for chronic disease risk reduction.

Dinner meal kits and a weekly grocery box containing foods that participants could use for lunch, breakfast and snacks were delivered by a commercial meal kit home delivery company specifically:

A box containing ingredients and recipes for 5 dinners. Participants had no choice in the meals that they received; these were selected by the meal kit company nutritionist as those meals that best met the Mediterranean Dietary pattern criteria. Participants were permitted to have some general dietary preferences that were taken into account, e.g. no pork. A fruit box containing approximately 15 pieces of seasonal fruit. A box containing foods for breakfast, lunch and snacks selected by the study nutritionists, namely cereal, yoghurt, eggs, wraps, rice, tuna, lentils, chickpeas, feta cheese, carrots, coleslaw, spinach, cucumber, tomatoes, muesli bars, nuts, pumpkin and sunflower seeds, and dried apricots, and included recipes and meal suggestions. Participants could not choose the contents of the box.

The diet was assessed against the PREDIMED diet criteria and met 11 out of 14 points [24]. The dietary pattern did not score points that would otherwise have been allocated for consumption of wine, sofrito (tomato sauce), four or more tablespoons of olive oil per day, and three serves of fish per week (two were provided in the weekly diet), but these were considered impractical in the context of the current study.

Participants were provided with dietary information that aligns with the NZ Ministry of Health Eating and Activity Guidelines [25], to guide their selection of non-provided foods that would make up their complete dietary intake. No other dietary counselling or behaviour change support was provided. Food was provided for the whole household regardless of how many participated in the study measurements.

### Outcome measures

The outcomes of this study were focused on feasibility questions for conducting a future trial.

1. Recruitment rates (time to recruit 30 participants, proportion of patients screened versus enrolled).
2. Proportion of household/whānau agreeing to enrol, average size of household/whānau.
3. MetSSS and other clinical parameters at baseline and 12 weeks. This included blood pressure, waist circumference, weight, height, body mass index (BMI), and body composition (bioimpedance), HbA1c and lipids.

Feasibility of the meal kit home delivery service; this included the utility of the distribution method and the acceptability of intervention for individuals and for household/whānau members. Fortnightly surveys were emailed to participants through the meal kit company using their Survey Monkey platform for the first 10 weeks of the study asking (i) if they had eaten all the food provided, (ii) what the reason was for not consuming items, and (iii) what changes would improve the meal kit for their household/whānau (supplementary file 1). The responses were compiled into a single document and inductive thematic analysis was carried out using NVivo (release 1.5.2 (946)). An informal exit interview was conducted at the 12 week visit where the participants were invited to reflect on the intervention and the food delivery. These comments were recorded and collated.

The relevant formulae used for calculating MetSSS were:

Male:

-5.4559 + (0.0125 × Waist Circumference (cm)) - (0.0251 × HDL (mmol/L) ×38.67) + (0.0047 × Systolic Blood Pressure) + (0.8244 × log(TAG (mmol/L) ×88.57)) + (0.0106 × Glucose (mmol/L) ×18)

Female:

-7.2591 + (0.0254 × Waist Circumference (cm)) - (0.0120 × HDL (mmol/L) ×38.67) + (0.0075 × Systolic Blood Pressure) + (0.5800 × log(TAG (mmol/L) ×88.57)) + (0.0203 × FPG1×18)

The multiplier preceding the clinical variable is the coefficient taken from the original publication for the MetSSS for non-Hispanic white and the multiplier after the clinical variable is the conversion factor from SI units to the units used in the original publication [26].

### Sample size and analysis

The main determinant of the sample size in this feasibility study was to enable reasonably precise estimation of the SD for the proposed primary outcome variable; the MetSSS, for a sample size calculation in a definitive future trial. A sample size of between 20 and 30 gives between 19 and 28 degrees of freedom to estimate the SD giving reasonable precision. Therefore the aim was to recruit 30 index participants.

The analysis for MetSSS was by a paired t-test and estimation of the confidence interval for the SD by an F-test. SAS version 9.4 was used.

## Results

### What are the recruitment rates for individuals to participate?

**Fig. 2:**
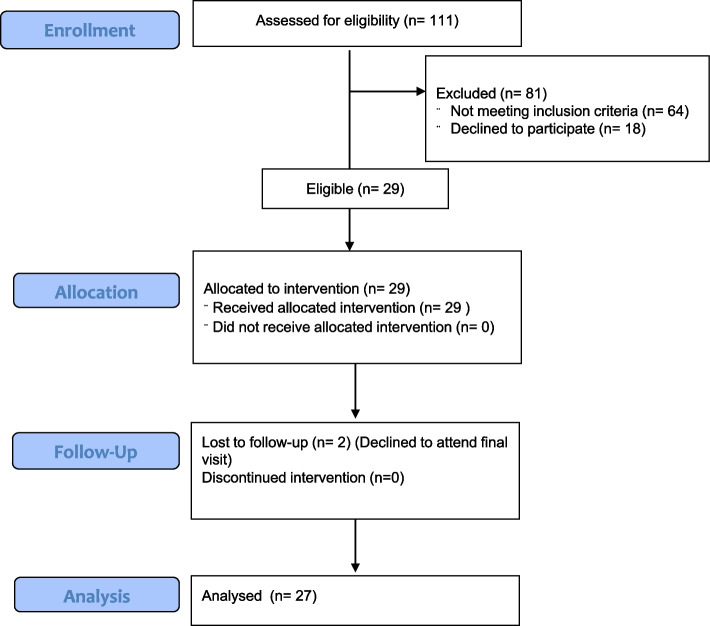
Consort participant flow diagram

One hundred and eleven people volunteered for the study, with 43 immediately classified as ineligible due to location or the participant declining to participate after reading the information sheet. Of the 68 people who completed the AUSDRISK questionnaire in step one of screening, 48 (71%) were eligible (AUSDRISK score ≥ 12) for the in person screening visit. Of those 48, all of whom presented for a screening visit, 29 (60%) met the MetSSS eligibility threshold (MetSSS > 3.5) and enrolled in the study (see Fig. 2. Participant Flow diagram). Their baseline characteristics are presented in Table 2. There were 20 index participants recruited at the Wellington Hospital (CEDOR) site and 9 at the Kōkiri Marae site. Recruitment was achieved over 12 weeks from October to December 2021, giving a rate of 2.4 index participants per week. Twenty seven participants completed the measurements after 12 weeks of the intervention. Two participants did not attend their final measurements despite having received all 12 weeks of food and were not included in the analysis of the MetSSS.

**Table 2:** Baseline characteristics of individuals included in the study

**INDEX**	**All**	**Kōkiri**	**Wellington Hospital**
**Variable**	**N/29 (%)**^**a**^	**N/9 (%)**^**a**^	**N/20 (%)**^**a**^
**Ethnicity**^**a**^			
Asian	2 (6.9)	0	2 (10)
European	5 (17.2)	0	5 (25)
Māori	16 (55.2)	9 (100)	7 (35)
Other	2 (6.9)	0	2 (10)
Pacific	4 (13.8)	0	4 (20)
**Sex**			
Male	7 (24.1)	1 (11.1)	6 (30)
Female	22 (75.9)	8 (88.9)	14 (70)
AUSDRISK **Score**^**b**^	15.6 (SD 3.5)	17.3 (SD 4.5)	14.9 (SD 2.8)
**Age**	50.7 (SD 11.0)	56.2 (SD 6.1)	48.3 (SD 11.9)
**HOUSEHOLD**	**All**	**Kōkiri**	**Wellington Hospital**
**Variable**	**N/50 (%)**^**a**^	**N/20 (%)**^**a**^	**N/30 (%)**^**a**^
**Ethnicity**^**a**^			
Asian	0	0	0
European	14 (28)	2 (10)	12 (40)
Māori	29 (58)	19 (95)	10 (33)
Other	6 (12)	2 (10)	4 (13)
Pacific	13 (26)	3 (15)	10 (33)
**Sex**			
Male	28 (56)	9 (45)	19 (63)
Female	21 (42)	10 (50)	11 (36)
Transgender	1 (2)	1 (5)	0
**Age**	33.9 (SD 16.6)	31.3 (SD 15.4)	35.6 (SD 17.4)

^a^participants may select more than one ethnicity

^b^AUSDRISK was the pre-screening tool used

### What is the acceptability and uptake of household/whānau members to be included in the study?

Fifty household/whānau members took part in the feasibility study; 28 males, 21 females and one transgender male, with a mean age of 34 y (Table 2). There were three children under the age of 11 years who agreed to participate, in two households/whānau. The ethnicities of household/whānau members paralleled the ethnicity breakdown of the index participants. The mean household/whānau size was 3.45 (SD 1.4) people and the mean number of people in a household/whānau who participated in the study was 2.84 (SD 1.2). Where there were household/whānau members who declined to participate in study assessments, food was provided for the whole household/whānau regardless. There were 11 households/whānau of 2–3 people, 14 of 4–5 and 4 household/whānau of 6. There were 33 household/whānau members who agreed to have anthropometric measures taken and of those 24 also consented to have blood taken. The remaining 17 completed the 24 h dietary recall only. No adverse events were reported in this study.

### Study challenges

There were some initial problems with online 24 h dietary recall whereby some households/whānau used the same ID number for each participant. It was more difficult to encourage all household/whānau members to attend for the final study visit compared to the baseline visit. Covid-19 provided an additional challenge and isolation meant that final visits were significantly delayed for the Kōkiri Marae site and some of the participants had completed the intervention and stopped receiving food for six weeks before their final visit could take place.

### *What is the mean and SD of each of the variables to be included in the primary *outcome* composite measure?*

The mean, standard deviation (SD) and change of the variables used in calculating the MetSSS for index participants, and the calculated score are all presented in Table 3. Table 4 presents the paired t-tests for change in selected variables over 12 weeks. A *per protocol* analysis was conducted (data not shown) but this did not change the outcome for MetSSS, although it did show a significant reduction in waist circumference.

**Table 3:** Data description of variables in the MetSSS for index participants

	**Mean (SD)**		
**Variable All (*N* = 27)**	**Baseline**	**3 Months**	**Change**
Waist Circumference (cm)	116.75 (15)	114.16 (13.16)	0.94 (5.72)
Fasting Plasma Glucose (mmol/L)	5.65 (0.75)	5.5 (0.81)	-0.1 (0.7)
Triglyceride (mmol/L)	2.04 (0.98)	2.16 (1.04)	0.11 (0.81)
HDL (mmol/L)	1.26 (0.31)	1.24 (0.28)	-0.04 (0.11)
SBP (mmHg)	135.55 (14.29)	137.8 (16.1)	3.59 (19.67)
MetSSS	1.05 (0.57)	1.03 (0.55)	0.03 (0.33)

**Table 4:** Paired t-tests for change in selected variables over 12 weeks

Variable All (*N* = 27)	Mean difference (95% CI)	*P*
BMI	-0.47 (-0.88 to -0.06)	0.027
MetSSS	0.03 (-0.11 to 0.16)	0.69
Weight (kg)	-1.37 (-2.62 to -0.11)	0.034
Waist circumference (cm)	-0.94 (-3.2 to 1.32)	0.40

### Dietary data

There were no statistically significant differences in any of the dietary intake variables from the 24 h dietary recall data between baseline and 12 weeks, however repeat data were only available for 18 participants.

### *What sample size is required for a future trial based on *MetSSS*?*

The estimate for standard deviation of the change from baseline MetSSS for all participants was 0.33 (95% CI 0.26 to 0.45). Table 5 shows total sample size required, based on the sum of two equally sized randomised groups, for various combinations of mean difference to detect and SD, based on the estimate from this study.

**Table 5:** Estimation of sample sizes required for a larger study based on MetSSS

MetSSS		N total and Power	
**Mean difference to detect**	**SD of change from baseline**	**80%**	**90%**
0.2	0.26	56	74
0.2	0.33	88	118
0.2	0.45	162	216
0.3	0.26	26	34
0.3	0.33	42	54
0.3	0.45	74	98
0.4	0.26	16	20
0.4	0.33	24	32
0.4	0.45	42	56
0.5	0.26	12	14
0.5	0.33	16	22
0.5	0.45	28	38

### Is it feasible and acceptable to create a meal kit dietary intervention and distribute this to individuals and their household/whānau for use in the main study?

The fortnightly surveys indicated that 40.4 (8.1) % of participants reported that their household/whānau consumed all the food provided each week. Thematic analysis identified participants frequently did not consume the muesli, coleslaw, and apples provided. Reasons stated for this were a combination of receiving too great a quantity of these foods (e.g. “*Too much muesli*”) and not liking them (e.g. “*apples becos i dont like em*”). The yoghurt was often not consumed due to being damaged during delivery and spilling (e.g. “*yoghurt because it was damaged on delivery*”). Many participants also stopped consuming the tuna wraps provided for lunch, as they became discouraged by the lack of variety in lunch options (e.g. “*getting sick of rice and tuna and slaw*”). Consequently, participants voiced wanting a greater variety of breakfast and lunch options (e.g. “*some variation to the breakfast/lunch box would be good*”). The comments relating to the “meal kit dinners suggested they were frequently eaten and enjoyed (e.g. “*We ate all the dinner meals provided and they were great*”).

The exit survey echoed these themes with all participants reporting they enjoyed the recipes and variety of dinner meals, but wanted more variety in breakfast and lunch options. The exit survey also highlighted ten participants were unsure how to prepare some of the food, with lentils, chickpeas and carrots being the most commonly reported unused foods for this reason. One family mentioned that the food did not fit with their (Samoan) culture. Participants also reported the quantity of vegetables supplied was overwhelming if they were not accustomed to preparing and consuming such quantities before participating in the study. Six participants identified an excess of food and feeling the need to consume it all to avoid wastage as an issue. These comments also echoed the fact the yoghurt and eggs were being broken regularly.

## Discussion

This feasibility study demonstrated that it is feasible to recruit participants and their household/whānau to participate in a dietary intervention where foods are delivered to participants using a meal kit home delivery service. Using a two-step screening protocol to identify individuals at cardio-metabolic risk enabled a recruitment rate which would be feasible for a future intervention trial. The AUSDRISK pre-screening questionnaire was easily conducted by the researcher over the telephone, zoom or via email communication and likely reduced time and resource burden for potential participants and research staff compared to having no pre-screening.

There have been four comparable trials to this feasibility intervention in the literature. Carman et al. (2021) undertook a smaller meal kit intervention, providing 3 meals per week for 6 weeks for low income families of 4 people, with high reported acceptability [27]. Utter et al., also in New Zealand, conducted two studies assessing the acceptability of meal kit interventions among families with adolescents. In the first study, ten families were recruited in 2016 from a school and provided with five dinners per week for eight weeks [28]. In the second, study nine families were recruited in 2019 from a youth clinic and provided with five meals per week for four weeks [29]. Intervention acceptability was high in both studies and positive findings included the quality of ingredients, ability to try new foods, easy-to-follow recipes, ease of preparation, adolescent involvement in cooking, experience of eating together, and perceived positive impact on nutrition. Oberle et al. (2020) investigated the acceptability of a meal kit with recipes and a grocery voucher to families in a weight management clinic [30]. Four families attended a focus group to explore their experiences with the meal kit, and reported convenience, portion size and grocery shopping guidance as the positive outcomes. The findings of these small studies are consistent with the current study in that they also demonstrated the feasibility of meal kit interventions. However the main focus of two of the studies was on food security [27, 29] and one study sought to support families to cook at home and eat together [28] and thus had a different lens to a metabolic health feasibility study.

This study has also demonstrated that it is feasible to use a commercial meal kit home delivery service to coordinate and distribute a NZ adapted Mediterranean dietary pattern to participants and their household/whānau for 12 weeks. Using a home delivery service overcame many logistical challenges which may have presented attempting to use supermarkets or other ways of providing participants with food. The approach was also positively received by participants. Importantly, this feasibility study identified changes to be incorporated into a larger future trial such as providing a greater variety of lunch meals, decreasing the quantities of certain foods provided, and resolving logistical challenges, such as the safe and unspoiled delivery of eggs and yoghurt. It also highlighted the need for additional participant support and education regarding, for example, how to use unfamiliar food items and overall meal portion size.

There was no statistical evidence of a change in MetSSS from baseline to 12 weeks, although there was a statistically significant decrease in both weight and BMI for the total group. A number of participants did not have their final measurements taken at 12 weeks, primarily because of Covid-19. Consequently, there was an interval of up to six weeks between the end of the food provision and the final measurements in these participants, which may have impacted the results.

Additionally, the delay to some of the final visits to the dietary pattern may have influenced the lack of a statistically significant change in the MetSSS as lower adherence to a dietary pattern can result in a smaller effect size [31, 32]. Adherence to the dietary pattern was not measured in this feasibility study, and it will be important to do so in a future trial, as seen elsewhere [33]. As the majority of households reported they did not consume all of the provided food on a weekly basis, this could crudely indicate suboptimal adherence, suggesting additional behaviour change support is warranted alongside food provision to enhance adherence to the dietary pattern. Systematic development of this additional support through using a behaviour change framework is worth considering to improve adherence in a future trial [34, 35].

This study successfully recruited participants and their whānau through a Māori health provider, Tū Kotahi, using their own staff, in alignment with their Kaupapa Māori framework – Whānau Tuatahi – which promotes research by and for Māori. This approach to health and wellbeing by and for their own people is an indigenous health model that links culture with health and self-determination [36]. A skilled indigenous Māori workforce embeds cultural values into service delivery and encourages Māori access into healthcare or, as in this project, supports participation in research for Māori. The partnership between CEDOR and Tū Kotahi resulted in the study being enriched for Māori participants and contributed to capability building for the Māori health workforce.

## Conclusion

It is feasible to recruit individuals and households/whānau to a NZ family-based dietary intervention, particularly in collaboration with a Māori health organisation. Using a two-step screening process minimised unnecessary participant and researcher time to identify participants meeting the inclusion criteria. Use of an established commercial meal kit home delivery service to provide food consistent with the intervention dietary pattern worked well with a few changes identified for any future trial. The feasibility study identified improvements to be made such as nutrition behaviour change support, more variety in food provided, more recipes and better matching of food quantity to family size. Logistical issues with assessment delays made it difficult to assess the full effect on MetSSS but there is a potential beneficial effect as seen by the decrease of two individual variables. However, the data collected have enabled a more accurate power calculation for a future trial.

### Supplementary Information

**Additional file 1.**

## Data Availability

Please contact the corresponding author for data requests.

## Abbreviations

BMI: body mass index
CHD: coronary heart disease
FBG: fasting blood glucose
g: gram
HbA1c: glycated haemoglobin
HDL-C: high-density lipoprotein cholesterol
HOMA-IR: homeostatic model assessment Insulin-resistance
LDL-C: low-density lipoprotein cholesterol
mmol/mol: millimoles per mole
REDcap: Research Electronic Data Capture
SF36 v2 NZ/Aust: Short form (36) health survey Version 2 NZ/ Australia
TC: total cholesterol
TGs: triglycerides
