# Supplementary material for: A high quality Aotearoa New Zealand dietary pattern adapting a Mediterranean diet for metabolic health: a feasibility study
Source: BMC Nutr. 2023 Dec 8;9:146. doi: 10.1186/s40795-023-00805-x (PMC10709956; doi:10.1186/s40795-023-00805-x)
Supplement: Supplementary file 1 — Additional file 1. [file 40795_2023_805_MOESM1_ESM.docx]

**Adherence Questions To be Asked Each Week**

"have you and your family managed to eat all the food provided" **Yes / No**

**If No**:  "Please list which foods/meals have not been eaten and if there is any reason for this (e.g. food preference, allergy etc) " **Long form answer**

AND

"which family members did not consume the provided foods and was there any reason for this (e.g. schedule, age, food preference)?" **Long form answer**
